# Vallecular Varix: A Perplexing Cause of Oral Cavity Bleeding

**DOI:** 10.5811/westjem.2015.10.28598

**Published:** 2015-12-08

**Authors:** Marc A. Polacco, Jacob Ossoff, Joseph Paydarfar

**Affiliations:** Dartmouth-Hitchcock Medical Center, Department of Otolaryngology, Lebanon, New Hampshire

## Abstract

Often discovered only after an extensive work up for hemoptysis and hematemesis, vallecular varices are a rare cause of oral bleeding that increase patient morbidity due to delay of diagnosis. We describe an 89-year-old male who presented with a week of intermittent oral blood production. A vallecular varix was identified on fiberoptic laryngoscopy after studies for hematemesis and hemoptysis had been performed, including negative esophagogastroduodenoscopy and bronchoscopy. Awareness of this pathology and key points in the patient history can direct the clinician toward the correct diagnosis, expediting treatment and limiting invasive diagnostic procedures for pulmonary or gastric etiologies of bleeding.

## INTRODUCTION

Blood discovered in the mouth most often originates from the upper airway, lungs, esophagus, or stomach. Due to difficulty of visual inspection, bleeding from the vallecula may be confused with hemoptysis or hematemesis. This case report details a vallecular varix, which can cause significant patient distress and morbidity as well as increased healthcare cost burden due to delay in diagnosis.

## CASE REPORT

The patient was an 89 year-old man who presented to the emergency department (ED) with one week of hemoptysis. He described that twice per day he would “feel warm liquid in my mouth, gag, and spit up clots of blood.” He denied a history of coughing, vomiting, or postnasal drip. His medical history was significant for coronary artery disease, hypertension, and chronic kidney disease. He was not taking anticoagulants. He had never smoked or consumed excess alcohol. His vitals on arrival included a temperature of 36.7 degrees Celsius, pulse of 82 beats per second, blood pressure at 135/64mmHg, respiratory rate 18 breaths per minute, and an oxygen saturation of 97% of room air. Laboratory data included hemoglobin 12.5gm/dL, platelets 131K/mm^3^, INR 1.0, PT 14.5 seconds. After obtaining a chest plain film with normal findings, a head, neck, and chest computed tomography was performed in the ED, which showed no evidence of masses, vascular malformations, or pulmonary embolism. Esophagogastroduodenoscopy showed mild duodenitis but no gastric ulcers or esophageal varices. After bronchoscopy failed to identify a source of bleeding, the patient was discharged as his symptoms seemed to have resolved during his hospitalization.

**Figure 1:**
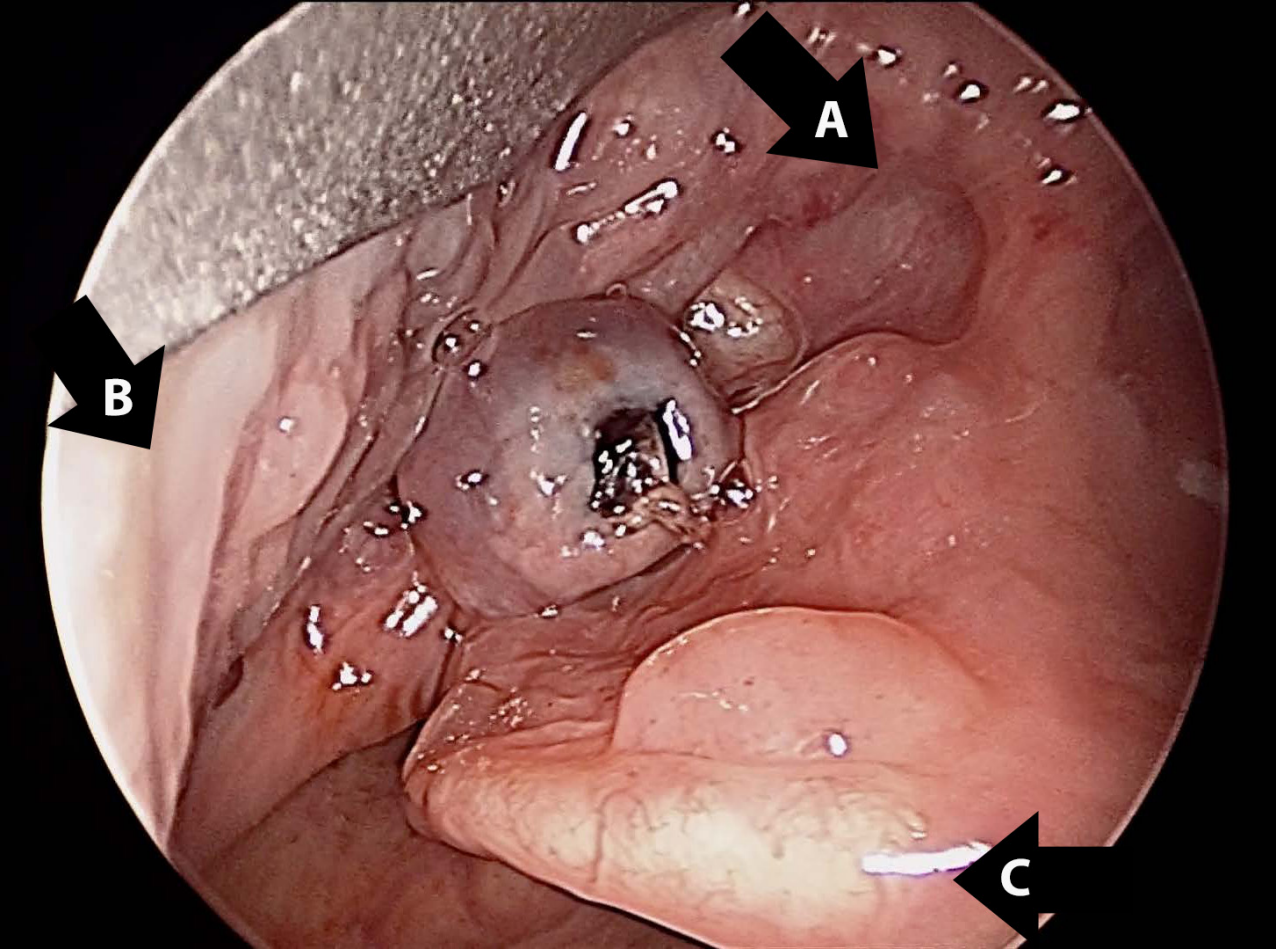
Vallecular varix. Pedunculated varix in the left vallecula with clot in center. A) Tongue base. B) Lateral pharyngeal wall. C) Epiglottis.

**Figure 2:**
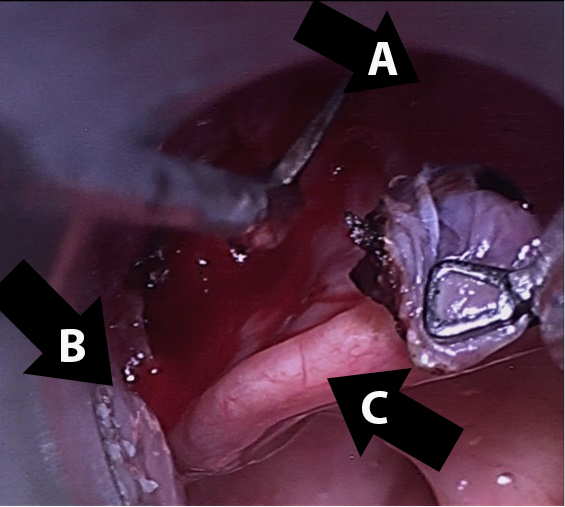
Extrication of varix. Extrication of varix at the base with Jako scissors after yttrium aluminum garnet laser cauterization. A) Tongue base. B) Lateral pharyngeal wall. C) Epiglottis.

Two days later the patient presented to the ED again with bleeding from the mouth. Otolaryngology was consulted and fiberoptic nasopharyngoscopy was performed, revealing normal nasal mucosa and no bleeding from the pharynx. However, upon asking the patient to protrude his tongue, a 0.5cm, non-bleeding, pedunculated varix was observed in the left vallecula. At this point the patient’s hemoglobin had decreased to 10.7gm/dL. The patient underwent direct laryngoscopy with microscope and yttrium aluminum garnet laser cauterization, followed by cold steel resection (Figure 1 and 2). The pathology report was varix with acute thrombus. The patient was discharged the next day after overnight observation for bleeding.

The patient’s post-operative course was complicated by a need to return to the operating room on post-operative day 10 due to bleeding from the surgical site. There was no evidence of recurrence of his varix and the bleeding was managed with suction cautery. Again, the patient was monitored overnight, demonstrated no further bleeding, and was discharged. The patient has remained well since.

## DISCUSSION

A patient presenting with blood in the mouth with no obvious source is a diagnostic puzzle as the etiology could be upper airway, pulmonary, or gastroesophageal. A detailed history is crucial in distinguishing which is the most likely anatomic location. Hemoptysis tends to present with cough, a history of lung disease, and frothy sputum, while hematemesis is frequently associated with nausea, a history of gastroesophageal disease, and frank or coffee-ground emesis.^1^ Bleeding from a nasal or anterior oral source is typically apparent on physical exam.

Bleeding from the vallecula, however, presents a particular diagnostic challenge, as it is neither common nor readily identified. Laryngoscopy is necessary, which may not be readily available in the ED setting and may require consultation. Moreover, it can present with hematemesis, hemoptysis, melena, or a combination of the three.^2^–^4^ This patient presented solely with gagging and sporadic pooling of blood in the back of his throat. Had the history guided intervention resulting in laryngoscopy first, the patient would have been spared tests that required radiation exposure and general anesthesia, in addition to repeated ED visits.

Unlike vallecular varices, lingual varices are more common in elderly populations, as well as those with a history of smoking and cardiovascular disease.^5^ It has been postulated that, like esophageal varices, portal hypertension is the underlying cause of varices at the base of the tongue.^6^ Although the patient in this case was advanced in age with a history of cardiovascular disease, he had no history of portal hypertension. Further studies are necessary to determine which patients are at greatest risk for this rare source of bleeding.

Vallecular varices are a rare, potentially life-threatening source of bleeding and should be considered in patients presenting with blood in the oral cavity without a history suggestive of pulmonary or gastroesophageal sources. Fiberoptic laryngoscopy is a fast, easily performed procedure that should be used early in order to prevent unnecessary testing and decrease healthcare costs associated with a delay in diagnosis.
